# The Effects of Online Text Comments on Patients’ Choices: The Mediating Roles of Comment Sentiment and Comment Content

**DOI:** 10.3389/fpsyg.2022.886077

**Published:** 2022-05-06

**Authors:** Jing Fan, Huihui Geng, Xuan Liu, Jiachen Wang

**Affiliations:** ^1^International Business School, Beijing Foreign Studies University, Beijing, China; ^2^School of Economic and Management, Beijing Polytechnic, Beijing, China; ^3^Business School, East China University of Science and Technology, Shanghai, China

**Keywords:** online health platform, online comments, text analysis, comment sentiment, comment content, patients’ choices

## Abstract

As an increasingly important application of mobile social media usage, online healthcare platforms provide a new avenue for patients to obtain and exchange information, referring not only to online doctor’s advice but also to the patients’ comments on a doctor. Extant literature has studied the patients’ comments facilitated with the direct numeric information gathered in the web pages including the frequencies of “thanks letter,” “flowers,” and “recommendation scores.” Adopting the text analysis method, we analyzed patients’ comments on the healthcare platform, focusing on the comments from two aspects, namely, comment contents and content sentiment. Based on the analysis of the data collected from one of the most popular healthcare apps named “Haodaifu” in China, the results show that the vast majority of the comments are positive, which basically follows the L-shaped distribution. Meanwhile, comment sentiment covering sentiment tendency and proportion of positive comments demonstrates significant effects on recent 2-week consultation by a doctor. One of the comment contents “patience explanation” has significant effects both on the total consultation and recent 2-week consultation by a doctor. The research findings indicate that the online preferences for and evaluations on doctors provide strong support and guidance for improving doctor-patient relationships and offer implications for medical practices and healthcare platforms improvement.

## Introduction

Research indicatesthat increasing population are seeking for medical health-related information on the Internet every day (Wallace et al., 2014; Hao, 2015; Mousavi et al., 2020). A survey ever found that 72% of Internet users have searched online for health-related information. Among those, one in five have browsed posted others’ comments on a particular disease or a doctor (Wallace et al., 2014). Online doctor comments were first studied and put into practice in the early 21st century. With advancement of information technology, online healthcare platforms (OHPs) gain rapid growth to meet up with the demands of medical services. How those comments posted on the healthcare platforms contribute to the improvement of peoples’ health, and platforms construction quality itself has become a spotlight of both researchers and practitioners.

In China, online health communities are mainly classified into three types, namely, online communication communities, professional-patient consultation platforms, and those as a medical part of social media platforms. Among them, haodf.com (Online Good Doctor) is one of China’s largest online health consultation platforms. Up to October 2021, Online Good Doctor has a history of over 10 years and has collected information on 860,000 doctors from 9,780 regular hospitals in China. Among them, 230,000 doctors have registered with their real names on the platform to directly provide online medical services for patients, and they have served more than 74 million patients. In terms of the data quality and matured operation modes provided by the platform, the study chose the platform as a reliable source for further empirical research.

Prior research has focused primarily on the factors that influence patients’ online decisions and behaviors including reputation (Liu et al., 2016), the response speed (Lu et al., 2019), login behavior (Chen et al., 2021), online review (Manchanda et al., 2015; Zeng and Guo, 2018; Marrero et al., 2020), and online rating (Gong et al., 2021). Some scholars also studied the influence of doctors’ personal attributes and online word-of-mouth on patients’ choice behavior (Hoffmann et al., 2014; Gao et al., 2015). Online comments in haodf.com involve a wide range of departments, and most of the reviews are positive (Hao, 2015).

Text analysis about online review has been mainly applied to shopping websites, books, movies, hotels, restaurants, and other social media platforms (Archak et al., 2011; Ghose and Ipeirotis, 2011; Yang et al., 2019). However, there is a paucity of research focusing on text analysis, in particular, online comment contents, patients’ expressions of emotions, and their influences on patients’ medical choice behaviors. Comment contents or review contents are utilized to present reviewers’ opinions, feelings, and preferences toward a particular product or service (Zhou et al., 2020). Online comments in the healthcare sector differ significantly in many aspects from online reviews of shopping. On the one hand, product reviews pay more attention to the quality of products, usage feedback information, and buyers’ feelings (Mudambi and Schuff, 2010). In contrast, online reviews for doctors focus more on the doctors’ skills and attitudes toward patients (Liu et al., 2020). On the other hand, patients are more sensitive and cautious about the online reviews than average products buyers. Patients and doctors usually have a stakeholder relationship (Guo et al., 2017), which requires long-term communications and differs significantly from the relationship between sellers and buyers. These differences indicate that reviews expressed in the OHPs, and their impacts on subsequent behaviors are quite different from product reviews.

For the content of online comment, a typical product comment contains two types of information, namely, the numerical rating and the review text (Li X. L. et al., 2019). The former is a quantitative summary of the reviewer’s experiences, attitudes, opinions, or sentiments toward a product or service, usually expressed as number of stars, and the latter is an open-ended textual description of the reviewer’s opinions toward the product or service (Li X. L. et al., 2019). Chen H. et al. (2020) studied 499 newspaper articles through automated content analysis, and people’s sentiment, national command and local response, government assistance, and postcrisis tourism product were identified among nine key themes. Therefore, this research aims to investigate how the key themes of patients’ comments affect patient’s choices on online healthcare community.

For the sentiment of online comment, it can be categorized into two categories, namely, positive and negative; or described with specific scores, e.g., very good, good, satisfactory, bad, and very bad (Prabowo and Thelwall, 2009; Li X. L. et al., 2019; Chen L. L. et al., 2020; Ali et al., 2021). However, there is a paucity of sentiment analysis applied to online healthcare literature researching user’s attitudes embedded in online comments (Lantzy and Anderson, 2020).

To advance our understanding of the online health contents and emotion labels of the doctor’s online reviews and their influence on patients’ choice of doctors, we combined the methods of qualitative and quantitative analysis by adopting archival data and taking insights from an exploratory online mining data to answer two research questions.

Question 1: How do we classify patients’ comment contents on a doctor? Are these comments positive or negative in sentiment?

Question 2: How do the comment classification and comment sentiment affect other patients’ choices?

In the current study, we collected about 10,050 comments on 201 doctors from 9 departments of 10 top-level hospitals in five provinces and cities. After processing, 8,285 comments on 190 doctors were finally chosen to analyze the content classification and content sentiment of online reviews in an OHP and their influences on patients’ choice. Research results suggest that both the comment sentiment and comment content of “patience explanation” significantly influence patients’ recent choices. Comment content of “patience explanation” also significantly influences patients’ choices in a long run. The research findings enrich the theoretical basis for online patients’ choices from the view of text analysis. More importantly, the demonstrated online preferences for and the evaluations on doctors provide strong support and guidance for improving doctor-patient relationships and offer insights to improve the quality of medical services and OHPs.

## Literature Review and Theoretical Model

### Online Healthcare Communities and Patients’ Choices

The OHP is a health consultation platform through which communications between doctors and patients occur. Except for the functioning of consultation with doctors, the online platform also functions as an important community for patients to collect and share health information to gratify their specific needs. On this platform, people can communicate about their symptoms, share medical information (Guo et al., 2018), and seek professional medical suggestions and knowledge about their diseases when they interact with online doctors (Wu and Lu, 2016; Gong et al., 2021). Meanwhile, patients can exchange with other patients suffering from the same or similar diseases on the platform to get emotional support. More importantly, patients can comment on the doctors they have consulted and the services they have experienced on the platform, which also serves as one of the reliable references for other patients to make decisions (Lu and Wu, 2016).

Unlike consultation offered by offline hospitals, online doctors are from different parts of professional institutions all over the country, which provides patients with more opportunities to choose their preferred doctors on the platform. In this regard, patients demonstrate more willingness to be involved in seeking relevant online information about a doctor’s professional specialty, skills, and service attitude so as to make their best decision (Karimi and Wang, 2017; Chen Q. et al., 2020). There are many indexes indicating doctor’s professional ability and popularity provided in the platform including “total number of consultations,” “number of consultations in recent 2 weeks,” “number of likes by the patients,” “number of flowers by the patients,” and “consultation prices.” All these indexes are reflected directly in the form of data illustrated in the website pages.

With regard to those indexes available on the OHP, scholars may use them as antecedent factors influencing a patient’s consulting intention and online follow-up intention toward a doctor as well as patient-doctor relationship (Cao et al., 2017; Li Y. F. et al., 2019; Gong et al., 2021). And some scholars use them, the number of online consultations for example, as the dependent variables affecting online doctor choices. However, less research has managed to co-relate those indexes and patients’ online mouth-of-word of patients.

To address the above gaps, the study adopts the online reviews as the antecedent factors influencing patients’ choices. Meanwhile, the total number of online consultation and 2-week number of online consultations are used to measure the patients’ choices including online appointment, online consultation, and telephone consultation.

### Text Analysis of Online Comments

Online comments have become increasingly critical to customer purchase decisions (Hong et al., 2017). Moreover, considerable studies have been conducted to analyze the text of online comments from different perspectives. In general, text analysis of online comments is mainly applied to the business operations of retailing, entertainments, tourism, and hospitality (Archak et al., 2011; Ghose and Ipeirotis, 2011; Yang et al., 2019). Huang et al. (2015) focused on the numeric information of the reviews (review rating and review length), while Salehan and Kim (2016) investigated the text-special characteristics (review sentiment and review complexity). Moreover, some research has also explored the effects of review volume, and readability on sales in various websites (Dellarocas et al., 2007; Baek et al., 2012; Yang et al., 2016; Sharma and Aggarwal, 2021).

#### Comment Sentiment

In the business research, sentiment analysis refers to the process of identifying different emotions (i.e., positive, negative, and neutral) toward a product or service in the text using computer-aided sentiment detection tools (Ding et al., 2021). The past few years have witnessed an explosion of research interest in the sentiment analysis field (Dashtipour et al., 2016), and it has been widely used in many fields (Koren et al., 2021; Li et al., 2022). Sentiment tendency is a common indicator for researching online reviews and online posts. Prabowo and Thelwall (2009) combined rule-based classification, supervised learning, and machine learning into a new combined method to test movie reviews, product reviews, and MySpace comments. NLP techniques, such as network analysis of the diffusion of content, sentiment analysis, and coding of prevalent themes, have been widely used in comment sentiment analysis (Chou et al., 2014). Previous research suggested that those reviews and posts are reflections of users’ emotions, which express to some extent how users feel positive, negative, or neutral toward services they have enjoyed (Cheng and Jin, 2019; Yang et al., 2019; Chi and Chen, 2021).

Despite of objectivity involved in online comments, scholars argued that those comments still exist as a form of subjective expressions of users’ emotions (Ghose and Ipeirotis, 2011; Guo et al., 2020). Statistics released indicates that emotional tendency expressed in online reviews tends to be J-shaped. Interestingly, research has also found that the majority of online reviews are positive and some are negative, but few reviews keep neutral stance (Hu et al., 2009; Guo et al., 2020). The possible reasons are twofold. First, online review posters are customers who have shopping experiences, and they are more likely to deliver positive reviews of products. This phenomenon is known as “purchase bias.” Second, some posters of positive reviews are influenced by consumers’ self-promotion motivation in a hope to maintain a good image in the public (Hu et al., 2009). In online healthcare, however, these two reasons may not be significant, but the J-shaped distribution is still widespread. This is because both hospitals and doctors encourage patients to express their reviews online, and there is a more intimate long-term dependent relationship between doctors and patients than the one between the buyers and sellers. In addition, doctors serving the online platforms are first chosen by the platforms according to their professional titles and educational background. As a result, as observed, the current online reviews cover a wide range of hospital departments and are mostly positive reviews (Hao, 2015). Due to the very limited negative reviews, the study sets a proportion of positive comments to total comments to reflect the negatives for the robust test.

#### Comment Content

Besides sentiment tendencies, the contents of comments were also considered in the study. Previous studies have shown that the contents of comments cannot be covered by sentiment tendencies (Archak et al., 2011). Moreover, the current artificial intelligence classification algorithm cannot work out the desired effect for the Chinese medical information classification, and the manual classification takes time in the case of a large number of reviews. Yang et al. (2019) first designed a new content framework, dividing users’ reviews on the company’s products into seven categories, and conducted research after classification with professional teams, insufficient research has been conducted to research the content classification index of online comments. Zhou et al. (2020) developed a research model of title-content similarity and sentiment consistency to test review helpfulness. Li X. L. et al. (2019) used a Joint Sentiment-Topic model to extract the topics and associated sentiments in review texts to investigate the influence of numerical and textual reviews on product sales performance.

In terms of techniques used to conduct content analysis, numbers of previous studies have used manifest variables as a proxy for message content factors (e.g., funny votes for customer enjoyment, review length for argument quality, and rating for valence) (Liu and Park, 2015; Kim et al., 2018). Recently, some researchers extracted content factors and other variables (e.g., comprehensiveness, relevance, clarity, and message valence) from the review text in a latent qualitative way (Srivastava and Kalro, 2019). Ding et al. (2021) examined positive and negative Airbnb reviews separately by using both Latent Dirichlet Allocation (LDA) and supervised LDA (sLDA) approach. In this article, we decided to use SnowNLP method to conduct content analysis.

### The Impact of Online Comments on Patients’ Choices

Pertaining to potential effects generated by the online comments on users’ behaviors, contents and sentiment of online comments have been identified as important antecedents (Chevalier and Mayzlin, 2006; Zhu and Zhang, 2010; Grabner-Kruter and Waiguny, 2015; Lu and Wu, 2016; Yang et al., 2019). For example, in the field of social networking sites, scholars have shown that the sentiment indicators and content types of online comments exert significant impacts on users’ participation behaviors. Similarly, social reviews about complaints receive more likes than those about price and quality issues (Yang et al., 2019). In the field of gaming e-commerce, studies have demonstrated that reviews generate significant impacts on users’ game purchase behavior and have a more significant impact on niche games played by users with network experiences (Zhu and Zhang, 2010). As for the existing literature on evaluation of doctors in healthcare field, limited research has confirmed basically that doctors’ service indicators, including professional indicators and procedural indicators, significantly affect patients’ experiences, thus influencing patients’ selection of doctors (Lu and Wu, 2016).

Different from traditional comment analysis, which mainly focuses on product attribution, we attempted to infer and extract the content and sentiment concept of patient’s comments on online healthcare community. As far as the comments in OHP are concerned, the content of online comments reflects the evaluation of patients on online doctors. Based on the text analysis, the content of the online comments is divided into three aspects, namely, timely reply, excellent medical skills, and consultation patience. In this article, the sentiment tendency of online comments reflects the degree of patients’ satisfaction with the doctor’s services. Based on the above statement, we contended that the comment sentiment of patients’ comments has a positive effect on patients’ choices.

**FIGURE 1:**
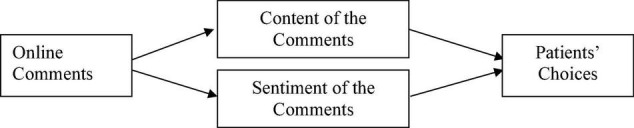
Proposed research model.

The theoretical model in this article is presented in Figure 1.”

## Materials and Methods

### Research Setting and Data

**FIGURE 2:**
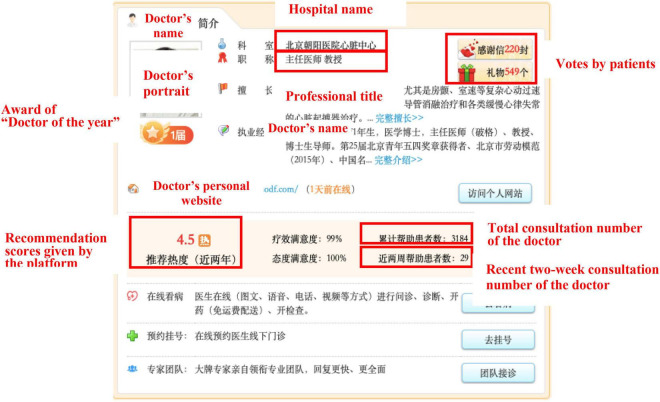
Doctor’s information page.

**FIGURE 3:**
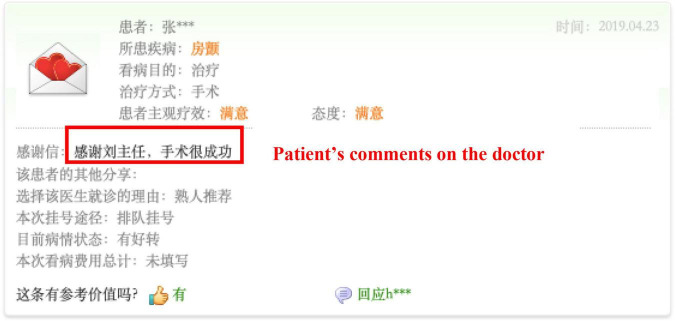
A patient’s comment page.

We selected the mobile app named Haodaifu as the current research context. We have three main reasons for selecting the popular app as our research target. First, as a mobile app, Haodaifu is a typical case of professional online health communities. Since its inception in 2006, Haodaifu has been viewed by more than 10 million patients and over 3,000 hospitals and 30,000 doctors have registered accounts in the platform. The reliable source of numerous users and comprehensive medical institutions provides sound evidence for the study. Second, Haodaifu mainly offers online consulting, telephone counseling, and mobile booking services for patients all over China. Meanwhile, Haodaifu provides patients with detailed background information of doctors and hospitals (e.g., titles, degrees, seniority, rank, the number of patients, and the comments by patients) and online generated information (e.g., patients’ comments, communication between doctors and patients, and medical advice) for reference (Zhang et al., 2017), which is presented in Figures 2, 3. Based on the evaluation on information above, patients can choose a trusted doctor, and the platform charges patients according to the types of services they choose. Therefore, Haodaifu has the data set for this study. Third, in this mobile app, patients who choose one of the doctor’s services can leave a message on the doctor’s personal homepage as comments. The comment contents and the patient’s basic information are displayed on the platform. This information presented in such a way that facilitates the comments’ collection work from all patients who were ever served by the same doctor in this study.

This article collected about 10,050 comments on 201 doctors from nine different departments in 10 first-class hospitals located in five provinces and cities in the app of Haodaifu from 2019. Hospital information can be seen in Table 1. For each doctor, we also collected their personal interface information, including their hospital and department information. After processing, 8,285 valid comments on 190 doctors were analyzed for the current research.

**TABLE 1:** Hospital information.

Region	Hospital	Department
Beijing	Beijing Chao-Yang Hospital	Cardiology Department Department of Respiratory and Critical Care Medicine Hematology Department Endocrinology Department Gastroenterology Department Orthopedics Department Urology Department Department of General Surgery Department of Vascular Surgery
	Chinese PLA General Hospital Peking Union Medical College Hospital	
Shanghai	Zhongshan Hospital Ruijing Hospital Huashan Hospital	
Wuhan, Hubei Province	Union Hospital	
Nanjing, Jiangsu Province	Jiangsu Province Hospital General Hospital of Eastern Theater Command	
Zhengzhou, Henan Province	The First Affiliated Hospital of Zhengzhou University	

### Measurement of Variables

#### Dependent Variable: Patients’ Choice

Patients’ choice is measured by two main dependent variables, namely, (1) the total consultation number of a doctor and (2) the recent 2-week consultation number of a doctor. Sought healthcare advice, online consultation, and telephone consultation are dominating determinants for patients to choose doctors. Therefore, the consultation number of a doctor is an important indicator to measure patients’ choice.

In this article, we mainly considered the online comments as the antecedent factors influencing patients’ choices. Meanwhile, the total number of online consultation and 2-week number of online consultations were used to measure the patients’ choices, which include online appointment, online consultation, and telephone consultation.

#### Independent Variables: Comment Sentiment and Comment Content

##### Sentiment Tendency of Online Comments

In the article, we used sentiment tendency of online comments to represent the comment sentiment. The sentiment tendency of online comments reflects the degree of patients’ satisfaction with the doctor’s services.

For sentiment tendency of online comments, we coded the variable by SnowNLP, which is a classic library in Python, aiming at processing Chinese text content. Since most of the natural language processing libraries are basically written in English, SnowNLP algorithm is inspired by TextBlob and mainly adopts Bayesian machine learning method to train text, which can easily process Chinese text content. For Chinese text, SnowNLP can process word segmentation, word tagging, sentiment analysis, text categorization, keyword abstraction, etc.

In the most situations, SnowNLP mainly focuses on online comments of shopping websites, which are not completely suitable for online healthcare reviews. Based on existing word library, we trained SnowNLP algorithm to become a sentiment analysis algorithm, which is especially suitable to analyze online healthcare review information.

In the first step, the trained algorithm was used to conduct sentiment analysis and obtain the score of sentiment tendency for the comments, in which one represents completely positive, zero represents completely negative, and 0.5 represents neutral. In the second step, to test the accuracy of the SnowNLP algorithm, we recruited 15 students to read those comments and re-evaluate the score of the sentiment tendency. Each comment was judged by five students. Those comments were deleted when the five students did not have a consistent opinion. Table 2 shows several examples.

**TABLE 2:** Examples of online comment’s sentiment tendency score.

Contents of online review	Sentiment tendency score
Dr. Cao is a young and middle-aged doctor with more than 10 years of rich clinical experience. He is knowledgeable and is able to combine theory and clinical practice well	0.999889866
It’s very convenient to register online	0.834978954986759
I got replies from the doctor and I was asked to call. I directly contacted by phone after seeing the doctor’s recommendation. It’s a pity that I wasted money on the medicine because the medicine did not function well. When I asked again, I was told to be hospitalized in Zhengzhou, and then there was no reply. Just want to ask a question, if a patient has any options to be treated well by the outpatient clinic, who would seek online diagnosis? Not to mention spending a lot of money, I was absolutely upset and can’t get a definite answer…	4.92626600179236E-07
I had bilateral varicose vein surgery, and went for a follow-up check 5 months after the surgery. Some detection indicators were not so good as before, and I was very disappointed! I felt that the previous diagnosis was too hasty. The doctor said that he did not know what was wrong when I went for a re-examination, so he prescribed some medicine and asked me to continue to take it	0.00231712325837108

##### Content Classification of Online Comments

In this article, the content of online comments mainly focuses on the patient’s evaluation about the doctors they have consulted. Chunyu online doctor platform, which is a comprehensive online professional healthcare platform, divides patients’ reviews on doctors into four aspects, including prompt reply, effective suggestions, good attitude, and clear explanation. Other scholars evaluate the doctors from two aspects, namely, technical level (treatment results) and procedural level (empathy, respect, and conscientiousness), and measure doctors’ services, respectively, from their professional skills and service attitudes (Lu and Wu, 2016).

We also calculated the cloud image of all 8,285 subject words of patient comments, as well as the cloud image of four randomly selected doctors (filtering out the personal information of doctors and hospitals). We found the words including “medical skills,” “medical ethics,” “thanks,” “surgery,” “patience,” and “superb” appear most frequently in the total comments. Accordingly, those words are also found to be high-frequency words in the cloud image of randomly selected doctors’ theme words.

In combination with other literature on the evaluation index system of doctors, we divided the online evaluation content of patients into three aspects, namely, timely reply, excellent medical skills, and patience explanation. Table 3 shows the definitions and examples.

**TABLE 3:** Definitions and examples of the content category framework.

Content categories	Definitions	Key words examples
Timely reply	It means that a doctor responds to the patient’s inquiries in time	Give prompt medical attention, answer all questions, quick response to inquiries, in time
Excellent medical skills	It refers to doctors’ good medical skills and effective treatment	Excellent medical skills, successful surgery, effective surgery, effective treatment on disease
Patience explanation	It means that the doctor treats the patient in a very good manner during the consultation process	Explain clearly, be patient and responsible, answer carefully, explain every detail

#### Control Variables

To avoid potential confound effects, the model in this article also contains the following control variables.

(1) Hospital information. Our data cover 10 hospitals located in five provinces. These hospitals are all Grade A hospitals in China. We set the dummy variable to indicate whether the hospital is in the municipality, which is directly under the jurisdiction of the central government. If the hospital is in the municipality directly under the administration of central government, it is 1; otherwise, it is 0.
(2) Doctor’s professional titles. The data for the study were collected from 190 doctors with professional titles include chief physicians, associate chief physicians, and attending doctors. The number of attending doctors in the sample was small. Therefore, we set a dummy variable to represent the professional title information of the doctor and whether the doctor is a chief physician or not.
(3) Recommendation scores generated by the app: a doctor’s rating score is displayed on the doctor’s recommendation score on the platform, which also affects the patient’s choice of doctor.
(4) Doctor’s honorable titles. “Doctor of the year” is an honorable title, which is awarded by the app and displayed on the doctor’s personal websites. The online health website presents this award to the top doctors each year, which is permanently visible on the doctor’s personal page. In this study, we have also controlled those honorable titles won by doctors to avoid the potential effects.
(5) Number of patient votes. The website displays the number of patient votes obtained by the doctor. The number is generated by patients who experienced online services and left a message or sent a thankful gift including “thanks letters” and “gifts” by the patients. Summary of variables are presented in Table 4. Table 5 shows the results of descriptive statistics.

**TABLE 4:** Summary of variables.

	Variables	Short	Definition
Dependent variables–patient’s choices	Total consultations by a doctor	Total	Accumulative consultation number by a doctor
	Recent 2-week consultations by a doctor	Recent	Consultation number in recent 2 weeks by a doctor
Independent variables–comment sentiment	Sentiment tendency	Sentiment	The scores of sentiment tendency of a patient’s comment which values from 0 to 1
Independent variables–comment content	Content1: timely reply	Cont1	Proportion of “timely response” comments received by the doctor
	Content2: excellent medical skills	Cont2	Proportion of “highly skilled” comments received by the doctor
	Content3: patience explanation	Cont3	Proportion of “patience explanation” comments received by the doctor
Control variables	Hospital location	Hos_Dummy	1 if the hospital is located in a municipality, otherwise is 0
	Job title of the doctor	Tit_Dummy	1 if the doctor is the chief physician, otherwise is 0
	Recommendation Score	RecScore	Scores recommended given by the app
	Doctor of the Year	YearDoc	Honorable titles of “Doctor of the year”, Number of year awards the doctor received
	Number of patient votes	Vote	Total number of patients voting for the doctor, including “thanks letters” and “gifts”

**TABLE 5:** Descriptive statistics.

Variables	Mean	SD	VIF	1	2	3	4	5	6	7	8	9	10	11	12
(1) Total	3,105	3,628		1											
(2) Recent	99.91	141.28		0.45	1										
(3) Sentiment	0.93	0.07	1.2	–0.12	–0.12	1									
(4) Positive	0.93	0.07	1.18	–0.11	0.12	0.99	1								
(5) Cont1	0.03	0.04	1.12	0.01	0.06	–0.09	–0.08	1							
(6) Cont2	0.59	0.18	1.11	–0.02	–0.14	0.02	0.02	–0.11	1						
(7) Cont3	0.12	0.1	1.09	0.04	0.1	0.06	0.06	0.2	–0.04	1					
Hos_Dummy	0.54	0.5	1.13	–0.05	0.05	0.13	0.11	0	0.15	0.2	1				
Tit_Dummy	0.53	0.5	1.14	0.22	–0.12	–0.31	–0.3	–0.02	–0.11	–0.06	–0.06	1			
RecScore	4.38	0.26	1.68	0.43	0.48	0.15	0.14	–0.13	–0.02	0.04	0.06	0.01	1		
YearDoc	0.41	0.88	1.74	0.63	0.5	–0.06	–0.06	0.1	–0.12	0.01	0.12	0.13	0.46	1	
Votes	130	145	1.96	0.76	0.5	–0.1	–0.09	–0.08	–0.08	–0.09	0.01	0.09	0.5	0.64	1

### Econometric Methods

Equations of Model 1 and Model 2 show the regression equation for the total consultations and recent 2-week consultations. Model 1 explains the relationship of total consultations and comment sentiment as well as comment content. The comment content includes three types (content 1: timely reply; content 2: excellent medical skills; and content 3: patience explanation). The others are control variables. Model 2 explains the relationship of recent 2-week consultations by a doctor and comment sentiment as well as comment content. The comment content includes three types (content 1: timely reply; content 2: excellent medical skills; and content 3: patience explanation). The others are control variables. Table 6 shows the explanation of econometric Model 1 and Model 2.

**TABLE 6:** The explanation of econometric model 1 and model 2.

	Model 1	Model 2
Dependent variable	Total consultations by a doctor (short as *Total*)	Recent 2-week consultations by a doctor (short as *Recent*)
Independent variables	Content1: timely reply (short as *Cont1*)	Content1: timely reply (short as *Cont1*)
	Content2: excellent medical skills (short as *Cont2*)	Content2: excellent medical skills (short as *Cont2*)
	Content3: patience explanation (short as *Cont3*)	Content3: patience explanation (short as *Cont3*)
	Sentiment tendency (short as *Sentiment*)	Sentiment tendency (short as *Sentiment*)
Control variables	Hospital location (short as *Hos_Dummy*)	Hospital location (short as Hos_*Dummy*)
	Job title of the doctor (short as *Tit_Dummy*)	Job title of the doctor (short as *Tit_Dummy*)
	Recommendation Score by App (short as *RecScore*)	Recommendation Score by App (short as *RecScore*)
	Doctor of the Year (short as *YearDoc*)	Doctor of the Year (short as *YearDoc*)
	Number of patient votes (short as *Vote*)	Number of patient votes (short as *Vote*)

Model 1:


(1) $$\begin{array}{c} LnTotal=\beta_{i,,\text{1}}Hos\_Dummy+\beta_{i\text{2}}Tit\_Dummy+\beta_{i3}RecScore\\ +\beta_{i4}YearDoc+\beta_{i,5}LnVote+\beta_{,i6}sd\_sentiment+\beta_{i,7}cont1\\ +\beta_{iti8}cont2+\beta_{i9,}cont3+\varepsilon \end{array}$$

Model 2:


(2) $$\begin{array}{c} Ln\left(1+Recent\right)=\beta_{i1,}Hos\_Dummy+\beta_{,i2}Tit\_Dummy\\ +\beta_{\,\,i3}Recscore+\beta_{i4}YearDoc+\beta_{i5}LnVote+\beta_{i6}sd\_sentiment+\\ \beta_{i7}cont1+\beta_{i8}cont2+\beta_{i9}cont3+\beta_{i10}LnTotal+\varepsilon \end{array}$$

To keep the data stationarity and normal distribution, we made the following variable transformation. First, we replaced the dependent variables of total consultations and recent 2-week consolations with their logarithmic forms. Second, because some doctors had no recent 2-week consultations, which means the variable value is 0, we used (1 + recent 2-week consultation) as the replaced variable. Third, for the variable “sentiment tendency,” we used its standardization form.

The results of OLS regression were shown in Tables 7 and 8. According to the adjusted R2 and F values, the independent variables in this article explained the dependent variables well. Meanwhile, VIF values of all variables are less than 4, indicating that collinearity problem is not a big issue.

**TABLE 7:** Content sentiment and content classification on the total consultation.

Variables	(1)	(2)
Sd_Sentiment		0.136
Cont1 (timely reply)		–0.421
Cont2 (excellent medical skills)		–0.369
Cont3 (patience explanation)		1.363**
Hos_Dummy	−0.159*	−0.191*
Tit_Dummy	0.385***	0.390***
RecScore	0.181	0.146
YearDoc	0.215***	0.195***
Ln(Vote)	0.706***	0.745***
ΔR2	0.6596	0.6773
ΔF	74.23***	45.08***

***p < 0.001, **p < 0.01, *p < 0.05.

**TABLE 8:** Content sentiment and content classification on the recent 2-week consultations.

Variables	(3)	(4)
Sd_Sentiment		0.206*
Cont1 (timely reply)		0.254
Cont2 (excellent medical skills)		0.002
Cont3 (patience explanation)		2.091*
Hos_Dummy	–0.215	−0.354 +
Tit_Dummy	−0.627***	−0.457*
RecScore	1.961***	1.775***
YearDoc	0.399**	0.421**
Ln (Vote)	–0.361	–0.217
Ln (Total)	0.391*	0.291 +
ΔR2	0.303	0.3255
ΔF	14.73***	10.12***

***p < 0.001, **p < 0.01, *p < 0.05, and ^+^p < 0.10.

## Results

### Main Results

#### Content Sentiment and Content Classification on the Total Consultation

For the total consultation, as shown in Table 7, regression (1) and (2) demonstrated the impacts of content classification and content sentiment of online comments on the total consultation number by a doctor. The comment of “patience explanation” has a significant positive impact on the total consultation number by a doctor (β = 1.363, *p* < 0.01). However, the other two kinds of comments exerted no significant effects on the total consultation number by a doctor. Meanwhile, the effect of sentiment tendency was positive, but it was not significantly associated with the total consultation.

The professional title of doctors has a significant positive effect on the total consultation of a doctor (β = 0.390, *p* < 0.001), and the region where the hospital is located had a significant negative effect on the selection of patients (β = -0.191, *p* < 0.05). The numbers of “Doctor of the year” awarded by the platform (β = 0.195, *p* < 0.001) and the number of votes by patients (β = 0.745, *p* < 0.001) both significantly affect the total consultation by a doctor.

#### Content Sentiment and Content Classification on Recent 2-Week Consultations

For the dependent variable of recent 2-week consultation, regression (3) and (4) showed the impact of content sentiment and content classification and online comments on the recent 2-week consultation of the doctors. It is shown that content of “patience explanation” has a significant positive effect on the recent 2-week consultation (β = 2.091, *p* < 0.05), while the other two types of comment have no significant effect. The sentiment tendency (β = 0.206, *p* < 0.05) has a significant influence on recent 2-week consultation by a doctor.

Further, doctors’ recommendation scores (β = 1.775, *p* < 0.001) and the influence of hospital titles both exert positive effects on patient selection (β = -0.457, *p* < 0.05). So does the award numbers of “doctor of the year” (β = 0.421, *p* < 0.01). Meanwhile, the total consultation has a positive relationship with the recent 2-week consultation by a doctor (β = 0.290, *p* < 0.05).

By comparing Tables 7 and 8, we found the difference of the influencing factors between the two dependent variables of total consultation and recent 2-week consultation. For the content classification, the third type of content, patience explanation, has significant positive effects on both of them but has a greater effect on the total consultation of a doctor, while the other two types of comments demonstrate insignificant effect. For the content sentiment, the sentiment tendency has significant effects on recent 2-week consultation but exerts no significant effect on the total consultation of a doctor. In addition, the total consultation also significantly affects the recent 2-week consultation of a doctor.

### Robustness Check

For the robustness test, we used the proportion of positive comments (short as positive) as an alternative variable of sentiment tendency. In the distribution of patients’ online comments on a random group of six doctors, we found that different from the J-type comment distribution of comments on traditional e-commerce products, patients’ online comments mainly remained positive, while negative comments are fewer than the ones on traditional e-commerce products and tend to be distributed in a reverse-L way. The research finding keeps consistent with the previous research on the overall online health community comments (Hao, 2015).

Equations of Model 3 and Model 4 show the new regression equation for the total consultations and recent 2-week consultations. Model 3 explains the relationship of total consultations and comment sentiment as well as comment content. The comment content includes three types (content 1: timely reply; Content 2: excellent medical skills; and content 3: patience explanation). The others are control variables. Model 4 explains the relationship of recent 2-week consultations by a doctor and comment sentiment as well as comment content. The comment content includes three types (content 1: timely reply; content 2: excellent medical skills; and content 3: patience explanation). The others are control variables. Table 9 shows the explanation of econometric Model 3 and Model 4.

**TABLE 9:** The explanation of econometric model 3 and model 4.

	Model 3	Model 4
Dependent variable	Total consultations by a doctor (short as *Total*)	Recent 2-week consultations by a doctor (short as *Recent*)
Independent variables	Content1: timely reply (short as *Cont1*)	Content1: timely reply (short as *Cont1*)
	Content2: excellent medical skills (short as *Cont2*)	Content2: excellent medical skills (short as *Cont2*)
	Content3: patience explanation (short as *Cont3*)	Content3: patience explanation (short as *Cont3*)
	Proportion of positive comments (short as *Positive*)	Proportion of positive comments (short as *Positive*)
Control variables	Hospital location (short as *Hos_Dummy*)	Hospital location (short as Hos_*Dummy*)
	Job title of the doctor (short as *Tit_Dummy*)	Job title of the doctor (short as *Tit_Dummy*)
	Recommendation Score by App (short as *RecScore*)	Recommendation Score by App (short as *RecScore*)
	Doctor of the Year (short as *YearDoc*)	Doctor of the Year (short as *YearDoc*)
	Number of patient votes (short as *Vote*)	Number of patient votes (short as *Vote*)

Model 3:


(3) $$\begin{array}{c} LnTotal=\beta_{i1}Hos\_Dummy+\beta_{i2}Tit\_Dummy+\beta_{i3}Rescore\\ +\beta_{i4}YearDoc+\beta_{i5}LnVote+\beta_{i6}positive+\beta_{i7}cont1+\beta_{i8}cont2\\ +\beta_{i9}contt3+\varepsilon \end{array}$$

Model 4:


(4) $$\begin{array}{c} \mathrm{Ln}\left(1+Recentt\right)=\beta_{i1}Hos\_Dummy+\beta_{i2}Tit\_Dummy\\ +\beta_{i3}Recscore+\beta_{i4}tYearDoc+\beta_{i5}LnVote+\beta_{i6}positive+\beta_{i7}cont1\\ +\beta_{i8}cont2+\beta_{i9}cont3+\beta_{i10}LnTotal+\varepsilon \end{array}$$

The results are shown in Table 10, which are consistent with the previous research. Proportion of positive comments for a doctor has a significant effect on the recent 2-week consultation of a doctor (β = 2.865, *p* < 0.05) while exerts no significant effect on the total consultation. The third type of content “patience explanation” demonstrates significant effects on both the total consultation (β = 1.363, *p* < 0.01) and the recent 2-week consultation (β = 2.102, *p* < 0.05), while the other two types of content show no significant effect.

**TABLE 10:** Robustness test.

Variables	(5)	(6)
Positive	0.245	2.865*
Cont1 (timely reply)	–0.418	0.186
Cont2 (excellent medical skills)	–0.369	–0.002
Cont3 (patience explanation)	1.362**	2.102*
Hos_Dummy	−0.191*	−0.346 +
Tit_Dummy	0.392***	−0.464*
RecScore	0.143	1.790***
YearDoc	0.195***	0.422**
Ln (Vote)	0.746***	–0.221
Ln (Total)		0.290 +
ΔR2	0.6774	0.3243
ΔF	45.1***	10.07***

***p < 0.001, **p < 0.01, *p < 0.05, and ^+^p < 0.10.

## Discussion

### Main Findings

Prior research has studied on the factors that influence patients’ online decisions and behaviors including reputation, the response speed, online review, online rating, and online word-of-mouth. However, there is a paucity of research focusing on text analysis, in particular, online comment content, patients’ expressions of emotions, and their influences on patients’ medical choice behaviors. The hypotheses have been reexamined in this study. The research findings suggest that not only the direct numeric information including the control variables (e.g., hospital locations, professional titles, patients’ votes) exerts effects on the patient’s choice but also the text information (e.g., the comment content) exerts significant effects on patient’s choices.

First, unlike online comments on traditional e-commerce products, which is the J-type review distribution, the vast majority of patients’ online comments remain positive according to our data presence. This may be due to the fact that both platforms and doctors encourage patients to make online comments, and the relationship between doctors and patients tends to be more intimate and last long-term than the one between online retailing buyers and sellers. Regarding the comment content classification, patients mostly commented on the doctor’s professional skills and service attitude, and few patients commented on the doctor’s “timely reply” classification (mean was 0.03). The words “medical skills,” “medical ethics,” “thanks,” “patience,” and so on are frequently displayed in the comments. Although “medical skills” and “medical ethics” are neutral terms in Chinese, “medical skills” often appears as “excellent medical skill” and “medical ethics” as “noble medical ethics” in the comments. As a result, we found that patients had more positive online comments on their doctors’ skills and attitudes, while they had fewer online comments about “timely reply.”

Our second finding is about the effects of content classification on the total consultations and the recent 2-week consultations by a doctor. The third type of comments in our research, patience explanation, has a significant positive effect on the recent 2-week consultations and demonstrates more significant effects on the total consultations by a doctor. The results suggest that patience explanation is an important indicator of patient choice. In other words, patients are more likely to choose a doctor who can answer their concerned questions patiently, which was different from the findings of previous research on doctors’ consultations. In the long run, the effect tends to be more significant. The research finding also keeps in line with previous studies (Lu and Wu, 2016). There are two possible reasons for insignificance of timely reply and excellent medical skills. First, because the data distribution is relatively concentrated, the differences between doctors are very small, which disables the variable to explain the dependent variable steadily. Second, the method of manual content classification could affect the accuracy of the results.

The third finding is about the effect of sentiment tendency on the total consultations and the recent 2-week consultations by a doctor. The sentiment tendency and the proportion of positive comments both exert positive effects on the recent 2-week consultations by a doctor but demonstrate no significant effects on the total consultations by a doctor. The results showed that the scores of sentiment tendency have a positive effect on the patients’ choice in the short term, which was roughly the same as previous research findings (Li Y. F. et al., 2019), but has a less effect on the patients’ choice in the long term. There are several reasons for this tendency. First, most of sentiment tendency scores about the doctors are very positive. The differences of sentiment tendency scores existing among doctors in a short term are relatively obvious, which produces significant impacts on a patient’s choices. However, in the long run, the sentiment tendency scores existing among doctors vary slightly, which is not a decisive factor for a patient’s choices. Second, when a user browses the online comments, it is very likely that he or she just browses the recent pages and neglects the comments delivered during a long-time span. In this vein, the comments in a long term, whether positive or negative, exert insignificant effects on the total consultation of a doctor. Third, sentiment tendency is an emotional indicator, which may stipulate people’s greater and immediate emotional resonance, and thus affects their choices in a short period, while the effect in a long-term incline is insignificant.

Finally, pertaining to the control variables which are direct numeric information shown in the web pages, we found that the influence of these factors varied over time. The influence of professional title of a doctor, the award number of “Doctor the Year,” and the hospital region on the total consultation of a doctor have more significant effects than that on the recent 2-week consultation. Patients’ votes have significant effects on the total consultation of a doctor but exert no significant effect on the recent 2-week consultation. Meanwhile, the total consultation of a doctor significantly affects the recent 2-week consultation.

### Theoretical Implications

In general, the study makes important contributions to the online comment literature and practices in the following aspects. First, from the text analysis view, this article explores the impact of online comments on patients’ choices in the healthcare platforms. In previous research, the majority has explored the antecedents influencing patients’ choices with the direct numeric information shown in the web pages. In our study, we combined natural language processing and manual processing to inventively divide online comments into the comment sentiment and comment content to represent the main variables.

Second, we further theorized and categorized the comments content into three types, namely, timely reply, excellent medical skills, and patience explanation, wherein the most significant content type is “patience explanation.” This means, compared with the face-to-face consultation in an offline hospital, the main purpose of online patients is to seek advisable information and guidance. In the online platform, they lay much emphasis on the doctor’s attitudes, and they are willing to more interactively communicate with doctors and get accurate information or suggestions. Meanwhile, in the current study, we enriched the emotional language library of Python with patients’ emotions on doctors by calculating the sentiment tendency index of each comment in the healthcare platform.

Third, it is interesting to note that the results show that those direct numeric variables (e.g., hospital region, doctor professional titles, and the number of awards won) have greater effect on the total consultation of a doctor. However, from the view of text analysis, the comment sentiment and comment content have greater effect on the recent 2-week consultation of a doctor than those on the total consultation by a doctor.

### Practical Implications

This study has several practical implications, from the perspectives of doctors, patients, and platform managers. For doctors, the result shows that patience explanation is an important indicator of patient choice. It is worth noting that the doctor can respond quickly to patients and give them enough time in good manner so as to maintain a good doctor-patient relationship during the online consultation. Moreover, the result shows that the scores of sentiment tendency have a positive effect on the patients’ choice in the short term but has a less effect on the patients’ choice in the long term. Doctors should value patients’ evaluation and maintain their reputation proactively. For patients, while receiving services offered by doctors on online community, they should take another perspective. Meanwhile, novel comments in OHCs may help patients simplify the information processing in decision-making, and they should be fully considered for further optimization as well. For managers, they should formulate appropriate regulations and encourage doctors to use online healthcare effectively and efficiently.

## Conclusion

Except for the contributions the study has made, this study also has limitations, which could be addressed by future research. First, all our samples are randomly selected from the first-class hospital because doctors in other hospitals have far fewer comments and consultations. With the development of OHPs, doctors from various regions and hospitals can join and welcome many more comments with new contents, positive or negative. For future study, we need to enlarge the sample dataset and take more text analysis. Second, in the app, communication contents between doctors and patients also contain a large amount of useful and important information. In the future research efforts, we can conduct in-depth analysis on these communication contents about patients’ sentiment and doctors’ patience and skills. In this regard, our research efforts not only contribute to literature on the consultation choices but also to practical improvement of the medical services quality.

## Data Availability Statement

The original contributions presented in the study are included in the article/supplementary material, further inquiries can be directed to the corresponding author.

## Author Contributions

JF contributed to conception and design of the study. JW organized the database. HG performed the statistical analysis. XL wrote the first draft of the manuscript. All authors contributed to manuscript revision, read, and approved the submitted version.

## Conflict of Interest

The authors declare that the research was conducted in the absence of any commercial or financial relationships that could be construed as a potential conflict of interest.

## Publisher’s Note

All claims expressed in this article are solely those of the authors and do not necessarily represent those of their affiliated organizations, or those of the publisher, the editors and the reviewers. Any product that may be evaluated in this article, or claim that may be made by its manufacturer, is not guaranteed or endorsed by the publisher.
